# Determination of the Thermodegradation of deoxyArbutin in Aqueous Solution by High Performance Liquid Chromatography

**DOI:** 10.3390/ijms11103977

**Published:** 2010-10-15

**Authors:** Chao-Hsun Yang, Yi-Shyan Chen, Jeng-Shiow Lai, Willy W. L. Hong, Chih-Chien Lin

**Affiliations:** 1 Department of Cosmetic Science, Providence University, 200 Chung-Chi Road, Shalu, Taichung, 43301, Taiwan; E-Mails: chyang@pu.edu.tw (C.-H.Y.); yishyan@gm.pu.edu.tw (Y.-S.C.); jslai@pu.edu.tw (J.-S.L.); 2 R&D Department, Denjelly Co., Ltd., 60 Jiabei 2nd St., Jhunan, Miaoli, 35058, Taiwan; E-Mail: willywlhong@gmail.com (W.W.L.H.)

**Keywords:** deoxyArbutin, hydroquinone, skin whitening, thermostability, tyrosinase inhibitor

## Abstract

Tyrosinase is the key and rate-limiting enzyme responsible for the conversion of tyrosine into melanin. Competitive inhibition of tyrosinase enzymatic activity results in decreased or absent melanin synthesis by melanocytes in human skin. DeoxyArbutin (4-[(tetrahydro-2H-pyran-2-yl)oxy]phenol), a novel skin whitening agent, was synthesized through the removal of hydroxyl groups from the glucose side-chain of arbutin. DeoxyArbutin not only shows greater inhibition of tyrosinase activity but is also safer than hydroquinone and arbutin. Hence, deoxyArbutin is a potential skin whitening agent for cosmetics and depigmenting drugs; however, stability of this compound under some conditions remains a problem. The lack of stability poses developmental and practical difficulties for the use of deoxyArbutin in cosmetics and medicines. Improving the thermostability of deoxyArbutin is an important issue for its development. In this research, we established an analytical procedure to verify the amount of deoxyArbutin in solutions using a high performance liquid chromatographic (HPLC) method. The results indicate that this novel skin whitening agent is a thermolabile compound in aqueous solutions. Additionally, the rate constant for thermodegradation (*k*) and the half-life (t_1/2_) of deoxyArbutin were determined and can be used to understand the thermodegradation kinetics of deoxyArbutin. This information can aid in the application of deoxyArbutin for many future uses.

## 1. Introduction

Although human melanin is the skin’s most important protection against the harmful effects of UV light, the dark skin caused by melanin accumulation is not considered cosmetically pleasing to most people [1–3]. The increased levels of melanin are also characteristic of a great number of skin diseases, including Melasma, Solar Lentigines, and Post-inflammatory Hyperpigmentation. Thus, there is an increasing desire for the development of skin whitening agents for both beauty and therapeutic purposes [4,5]. Tyrosine is the precursor for the synthesis of melanin. Tyrosinase is the key and rate-limiting enzyme responsible for the conversion of tyrosine into melanins by melanocytes in human skin [6,7]. Inhibition of the enzymatic activity of tyrosinase by competitive inhibitors results in decreased or absent melanin synthesis by the melanocytes in human skin [8,9].

**Figure 1:**
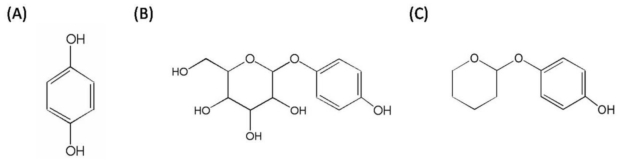
Chemical structure of hydroquinone (A), arbutin (B) and deoxyArbutin (C).

Many compounds that bind to the tyrosinase active site and inhibit melanin synthesis have been developed as agents to lighten skin, including hydroquinone, arbutin and deoxyArbutin (dA, Figure 1) [10]. Hydroquinone is the most conventional skin whitening agent. However, it has numerous unfavorable effects with long-term application, including irritative dermatitis, melanocyte destruction, contact dermatitis and ochronosis [11]. Arbutin is a natural glycosylated hydroquinone of the bearberry plant, and it is safer and less cytotoxic compared with hydroquinone. Although arbutin is quite safe, it inefficiently inhibited melanin production *in vivo* in several studies [12,13]. DeoxyArbutin (4-[(tetrahydro-2H-pyran-2-yl)oxy]phenol) was first reported by Boissy and his colleagues as a novel skin whitening agent [10,14]. This compound has been synthesized by the removal of hydroxyl groups from the glucose side-chain of arbutin (Figure 1), has greater inhibition of tyrosinase activity and is safer than hydroquinone and arbutin [13]. DeoxyArbutin also demonstrates fast and persistent skin lightening effects both in a hairless, pigmented guinea pig model and in human skin [11,13]. Hence, deoxyArbutin is a potential skin whitening agent for cosmetics and depigmenting drugs.

Arbutin produces the hydroquinone *in situ* upon absorption into skin, thus, it has potential instability and tends to change color due to oxidation at higher temperature in formulation [15]. Because deoxyArbutin is a derivative of arbutin, there is also a problem with compound stability under some conditions. This stability issue causes problems with the use of deoxyArbutin in cosmetic products and medical drugs. As a result, improving the thermostability of deoxyArbutin is an important aspect for its future application. This is the first report describing the thermostability of deoxyArbutin in an aqueous environment. In this study, we first established the analytical procedure to confirm the identity of the compound in solutions using a high performance liquid chromatographic (HPLC) method. Additionally, in order to completely understand deoxyArbutin stability, we investigated the degradation process of deoxyArbutin at several temperatures. The rate constant for the thermodegradation (*k*) and half-life (t_1/2_) of deoxyArbutin were also determined.

## 2. Results and Discussion

The major purpose of the work was to investigate the thermostability of deoxyArbutin in an aqueous solution. For this reason, the first part of the study examined the solubility and measured the UV spectrum of deoxyArbutin in an aqueous solution. Second, we established the analytical procedure to confirm the quantity of deoxyArbutin in solutions using an HPLC method. Using this method, the kinetics of deoxyArbutin thermodegradation were determined.

### 2.1. Preparation of DeoxyArbutin in Aqueous Solutions

Because the hydroxyl groups of the glucose side-chain are removed (Figure 1), deoxyArbutin is insoluble in water at room temperature; therefore, we used propylene glycol as the solvent to aid in the dissolution of deoxyArbutin in water. In our study, deoxyArbutin can be dissolved up to 13% (w/w) in propylene glycol and butylene glycol (BG) at room temperature (data not shown). Previous research has examined the solubility of deoxyArbutin in an ethanoic solutions, and the results showed that deoxyArbutin can be dissolved in a simple vehicle of propylene glycol-ethanol-water at a 1:2:1 (v/v/v) ratio or 1:1 (v/v) ethanol-water [13].

The United States Food and Drug Administration (FDA) has determined propylene glycol to be a Generally Recognized As Safe (GRAS) ingredient for use in cosmetics, food, and medicines. The World Health Organization (WHO) has also identified it as safe for use [16]. Similar to water, propylene glycol is commonly used in cosmetic products, mainly as a solvent or a humectant. It also helps to bind the various ingredients in the cosmetics. Although ethanol can be used to help dissolve deoxyArbutin in water, skin irritation caused by ethanol cannot be completely avoided for ethanol-containing cosmetic products [17]. For these reasons, we used propylene glycol as the solvent to aid in the preparation of the deoxyArbutin aqueous solutions.

### 2.2. Ultraviolet Spectrum of DeoxyArbutin

**Figure 2:**
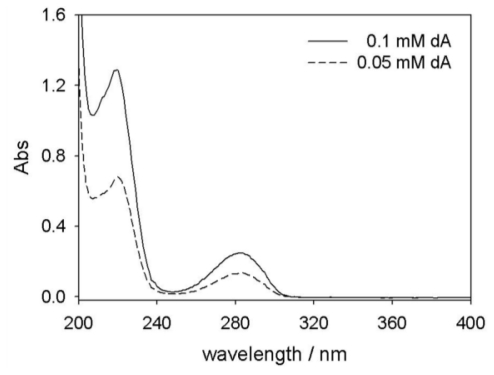
Ultraviolet spectrum of deoxyArbutin at the concentrations of 0.05 and 0.1 mM.

To establish an analytical method for deoxyArbutin, we used a UV-Vis spectrophotometer to collect the UV spectrum of deoxyArbutin aqueous solutions. Both 0.05 and 0.1 mM deoxyArbutin in deionized distilled water (ddH_2_O) with 10% propylene glycol were placed in a quartz cell and then scanned using a UV-Vis spectrophotometer between wavelengths from 200 to 400 nm. The results are shown in Figure 2. The absorbance spectrum of deoxyArbutin shows a minimum of 248 nm and two maximums of 232 and 283 nm. The absorbance levels increased with increasing concentration of deoxyArbutin (Figure 2).

Similar to arbutin and hydroquinone, deoxyArbutin has two maximum absorbances at 232 nm and 283 nm, which are close to 230 and 280 nm. These maximum absorbances of deoxyArbutin result from the structure of benzene chromophore. In previous research, the HPLC UV detector wavelength was set to 280 nm in order to simultaneously detect arbutin and hydroquinone in solutions [18]. Therefore, we choose a UV wavelength of 280 nm for the detection of both deoxyArbutin and hydroquinone, using the HPLC method.

### 2.3. HPLC Analysis of DeoxyArbutin

**Figure 3:**
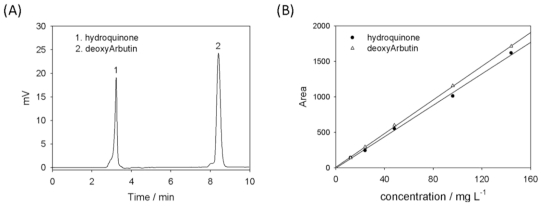
Chromatogram of the separation on a C18 column (A) and calibration curves for hydroquinone and deoxyArbutin (B).

Standard solutions (20 μL, 12–144 mg/L) of deoxyArbutin and hydroquinone were injected into an HPLC to establish the analytical method. The chromatogram, shown in Figure 3(A), demonstrates the separation achieved under these conditions and the elution order, which was identified by co-injection of the individual compounds at 24 mg/L. Figure 3(A) shows that hydroquinone and deoxyArbutin have retention times (RT) of 3.32 minutes and 8.45 minutes, respectively. Using a UV detector at 280 nm, the responses were linear for each compound for concentrations between 12 and 144 mg/L. In addition, least-squares regression analysis of the data for the two compounds resulted in good straight-line fits over the concentrations examined, with R^2^ greater than 0.995. Figure 3(B) shows the calibration graph obtained. HPLC analysis of deoxyArbutin and hydroquinone in aqueous solutions was achieved.

In several studies, hydroquinone and other compounds were separated using HPLC with a conditioned mobile phase such as methanol-water at 80:20 (v/v) and acetonitrile-water-formic acid at various ratio [18–21]. Thus, it is not difficult to separate a hydrophilic compound (hydroquinone) from a hydrophobic compound (deoxyArbutin) using an HPLC method according to previous studies with a slight modification. In the present study, we used a simple mixed mobile phase containing 60% methanol and 40% water to separate these two compounds within 10 minutes using HPLC (Figure 3). This established assay can help us to investigate the thermostability of deoxyArbutin in an aqueous solution. The quantity of hydroquinone produced could also be monitored using this method.

### 2.4. Thermodegradation of DeoxyArbutin and Accumulation of Hydroquinone

**Figure 4:**
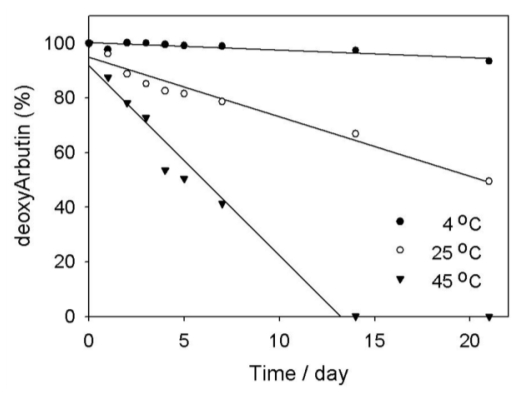
Thermodegradation of deoxyArbutin at various temperatures in an aqueous solution.

DeoxyArbutin (1 × 10^−4^ M) solutions in ddH_2_O (pH 7) and 10% propylene glycol were placed in glass bottles and kept in the dark. The solutions were then exposed to various temperatures in an incubator. The temperatures were 4 °C (low temperature), 25 °C (middle temperature) and 45 °C (high temperature). At each time point the samples were analyzed using the HPLC method, and the resulting deoxyArbutin retention percentages are shown in Figure 4. The results show that deoxyArbutin in an aqueous solution decomposed to the not detectable (N.D.) level when the solution was kept at 45 °C for 14 days (Figure 4). Moreover, deoxyArbutin at 25 °C also showed an obvious decrease from the first day (96.14%) to the 21st day (49.42%). Only at 4 °C did deoxyArbutin demonstrate stability in the solution (93.43% of retention) through the 21st day (Figure 4). Therefore, in an aqueous solution, deoxyArbutin is much more stable at low temperatures than that at high temperatures.

The stability of arbutin has also been studied in previous research. The results indicated that arbutin has a t_90%_ (the time necessary to obtain a decrease of 10% of the initial concentration) of 15.4 days at 20 °C [22]. Compared to arbutin, deoxyArbutin has a much shorter t_90%_ of about 1.9 days at 25 °C (Figure 4). Thus, it appears that deoxyArbutin is very thermolabile.

**Figure 5:**
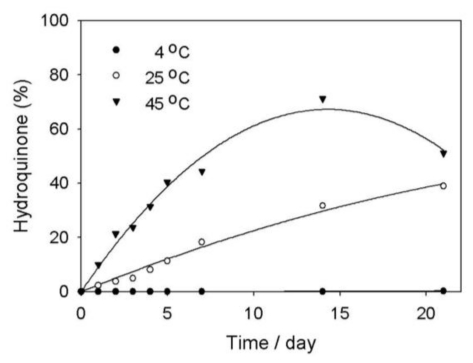
Accumulation of hydroquinone in the deoxyArbutin-containing solutions at various temperatures.

At each time point, we also checked the production of hydroquinone in the deoxyArbutin aqueous solutions. The results indicated that the amount of hydroquinone increased from the first day to the 21st day at every temperature condition (Figure 5). The hydroquinone concentration not only increased at the high temperature condition (45 °C) from the first day (9.92%) to the 21st day (51.08%) but it also increased in concentration at room temperature condition (25 °C). Additionally, the maximum increase of hydroquinone produced was 71.21% by the 14th day at 45 °C (Figure 5). It is notable that the amount of hydroquinone at 4 °C was below the N.D. level for all time points, except on the 21st day. The hydroquinone percentage at 4 °C on the 21st day was only 0.27% (Figure 5).

In earlier studies, the skin whitening efficacy and the cytotoxicity of deoxyArbutin and hydroquinone were examined [10,11,13,14], however, no reports have confirmed that deoxyArbutin may decompose to hydroquinone. Although it is not surprising that deoxyArbutin will hydrolyze to hydroquinone, this study is the first report that proves the degradation process using an HPLC method.

**Figure 6:**
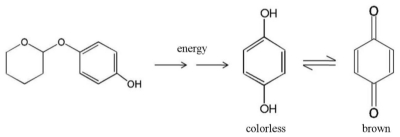
Scheme of a possible mechanism for deoxyArbutin decomposition in an aqueous solution to colorless hydroquinone and brown benzoquinone.

The possible decomposition mechanism of deoxyArbutin to hydroquinone is illustrated in Figure 6. In a high temperature aqueous environment, the free electrons may attack the oxygen atom in the backbone between the phenol element and the tetrahydro-2H-pyran part of deoxyArbutin (Figure 6). Consequently, deoxyArbutin may degrade to hydroquinone and other molecules such as tetrahydro-2H-pyran-2-ol in an aqueous solution. The produced hydroquinone (colorless in an aqueous solution) will oxidize to benzoquinone, which can turn the clear aqueous solution brown (Figure 6) [23,24]. To clarify the precise mechanism of deoxyArbutin thermodegradation, additional studies should be performed.

### 2.5. Kinetics of DeoxyArbutin Thermodegradation

To understand the kinetics of deoxyArbutin thermodegradation, we evaluated and calculated the quantities of deoxyArbutin retained at each time point. The rate constant for deoxyArbutin thermodegradation and the half-life of deoxyArbutin can help us obtain the exact characteristics for the decrease of deoxyArbutin concentration as a function of temperature.

Because the deoxyArbutin concentration decreases with first-order kinetics at the given temperature, the equation for the exponential decomposition curve (1) could be adopted to calculate the amount of deoxyArbutin remaining in the aqueous solutions:

(1) $${\text{C}}_{\text{t}}={\text{C}}_{\text{o}}{\text{e}}^{-\text{kt}}$$

where Ct and Co are the current (at day t) and the initial amount of deoxyArbutin in an aqueous solution, respectively. The *k* value is the rate constant for deoxyArbutin thermodegradation. The *k* value was obtained from the slope of the plots showing the percent of deoxyArbutin *vs*. time, as determined over the 21-day incubation period using the HPLC method. The half-life, t_1/2_, can be defined as the t value where C_t_/C_o_ = 1/2 and can be obtained from equation (2) as follows:

(2) $${\text{t}}_{1/2}=\text{ln}(1/2)/(-k)=0.693/k$$

The rate constant (*k*) was obtained at various temperatures from 4 to 45 °C and gave half-life (t_1/2_) values for deoxyArbutin as shown in Table 1. The *k* values for deoxyArbutin thermodegradation were 0.0037, 0.0312 and 0.0947 at 4 °C, 25 °C and 45 °C, respectively (Table 1). The t_1/2_ values for deoxyArbutin were 186.07, 22.24 and 7.11 days at 4 °C, 25 °C and 45 °C, respectively (Table 1). Therefore, deoxyArbutin may quickly degrade to other substances (including hydroquinone) at 25 °C and 45 °C in and aqueous solution (Table 1). These results indicate that the thermostability of deoxyArbutin is relatively poor.

In the former study, the t_1/2_ value of arbutin at 20 °C was about 11 weeks (77 days) in an aqueous solution [22], consequently, results indicated that arbutin is very thermolabile. However, the t_1/2_ value of deoxyArbutin at 25 °C in an aqueous solution is only about one week (7.11 days) and is decidedly shorter than the t_1/2_ value of arbutin at 20 °C (Table 1). Thus, even if the temperature conditions were different between these two results, it is clear that deoxyArbutin is more thermolabile than arbutin in an aqueous solution.

## 3. Experimental Section

### 3.1. Materials

DeoxyArbutin was purchased from Denjelly Co., Ltd (Miaoli, Taiwan, R.O.C). Hydroquinone was purchased from Wako Pure Chemical Industries (Osaka, Japan). HPLC-grade methanol was purchased from Merck (Darmstadt, Germany). Analytical grade propylene glycol and other chemicals were purchased from Sigma-Aldrich (St. Louis, MO, USA). Deionized distilled water (ddH_2_O) for solutions and buffers was obtained using a Milli-Q system (Millipore, Bedford, MA, USA).

### 3.2. Ultraviolet-Visible (UV-Vis) Spectrophotometer Analysis

To overcome the low solubility of deoxyArbutin in pure water, deoxyArbutin was initially dissolved in propylene glycerol then mixed with ddH_2_O. DeoxyArbutin was dissolved in ddH_2_O with 10% propylene glycol at 0.05 and 0.1 mM and then analyzed using a T60 UV-Vis spectrophotometer (PG Instruments, U.K.). The UV wavelength was set to scan from 200 to 400 nm and the scanning spectral bandwidth was 2 nm with a scanning speed of 200 nm/minute. The resulting UV spectrum was analyzed with the UVWin5 Software (PG Instruments).

### 3.3. High Performance Liquid Chromatography (HPLC) Analysis

The 20-μL samples of the deoxyArbutin solution were injected into an HPLC (Agilent 1100 series, USA). A pre-packed C18 reversed-phase column (Mightysil RP-18, GP 250-4.6, Kanto Chemical, Tokyo, Japan) was used. The HPLC UV detector wavelength was set to 280 nm in accordance with the UV-Vis Spectrophotometer analysis results (Figure 2). The mobile phase was composed of methanol-water (60:40 (v/v), pH 7) and run at a flow rate of 1 mL/minute. The solvents were filtered separately through a 0.45-mm filter (Millipore) and mixed in the desired proportions. Standard solutions of deoxyArbutin and hydroquinone (12–144 mg/L) were analyzed using the previously described HPLC method. The UV detector wavelength was set to 280 nm for both deoxyArbutin and hydroquinone [18,21]. The ratio of the compound peak area to internal standard peak area was calculated using the corresponding concentrations to obtain the calibration graph.

### 3.4. Thermostability Studies

Solutions of deoxyArbutin (1 × 10^−4^ M) in ddH_2_O (pH 7) containing 10% propylene glycol were placed in glass bottles and kept in the dark. In an incubator, these samples were exposed to the following temperatures: 4 °C (low temperature), 25 °C (middle temperature) and 45 °C (high temperature). At select times, the solutions were analyzed using the previously described HPLC method to monitor the thermodegradation progression of the compound. The rate constant for thermodegradation (*k*) and half-life (t_1/2_) of deoxyArbutin were calculated according to standard first-order kinetics [25].

### 3.5. Statistical Analysis

All data were analyzed in three independent experiments and presented as means. Statistical comparison of means and simple correlation coefficients were performed using the Student’s *t*-test.

## 4. Conclusions

In summary, we established an analytical procedure to verify the amount of deoxyArbutin in solutions using an HPLC method. We also demonstrated that this novel skin whitening agent is a thermolabile compound in an aqueous solution. The rate constant for thermodegradation (*k*) and half-life (t_1/2_) data for deoxyArbutin can help us understand the thermodegradation kinetics of deoxyArbutin. In conclusion, we believe that these results may aid in the development of deoxyArbutin’s use for many applications, including cosmetics and medicines.

## Figures and Tables

**Table 1:** Rate constants for thermodegradation (*k*) and half-life (t_1/2_) values for deoxyArbutin in an aqueous solution.

Temperature (°C)	*k*	t_1/2_ (day)
4	0.0037	186.07
25	0.0312	22.24
45	0.0974	7.11
